# Syphilis Screening in Pregnancy During Emergency Department Gonorrhea and Chlamydia Testing

**DOI:** 10.5811/westjem.54849

**Published:** 2026-09-24

**Authors:** Michael P. Phelan, Elizabeth N. Dewey, Fredric M. Hustey, Stephen W. Meldon, McKinsey Muir, Janet Wu, Matthew J. Campbell, Oluwatosin Goje, Kamran Kadkhoda, Charles B. Foster

**Affiliations:** * Cleveland Clinic, Department of Emergency Medicine Main Campus, Cleveland, Ohio; † Cleveland Clinic, Center for Populations Health Research Quantitative Health Sciences, Cleveland, Ohio; ‡ Cleveland Clinic, Department of Pharmacy, Cleveland, Ohio; § Cleveland Clinic, Women’s Health Institute, Cleveland, Ohio; || Cleveland Clinic, Diagnostics Institute, Pathology and Laboratory Medicine Department, Cleveland, Ohio; # Cleveland Clinic, Section of Pediatric Infectious Diseases, Cleveland, Ohio

## Abstract

**Introduction:**

Congenital syphilis is rising in the United States, especially among infants born to women with limited prenatal care. Emergency departments (ED) offer opportunities for early detection, particularly among patients evaluated for urogenital sexually transmitted infection (STI) concerns. In October 2019, our health system implemented an ED STI order set that promoted HIV co-testing and included a preselected option for syphilis testing. In this study, we evaluated change in syphilis testing during the post-implementation period among ED encounters with both pregnancy and gonorrhea/chlamydia (GC/CT) testing.

**Methods:**

We conducted a retrospective, quasi-experimental study across 20 EDs, using health record data from 2018 to 2023 for patients aged 15–44 years who received both pregnancy and GC/CT testing. We measured annual syphilis and HIV testing and positivity, stratified by pregnancy status. The primary study outcome was syphilis testing during the encounter; HIV testing and detection outcomes were secondary.

**Results:**

Among 59,171 ED encounters with pregnancy and GC/CT testing, 5,178 (8.8%) involved pregnant patients, and 9,538 (16.1%) received syphilis testing. Syphilis testing among pregnant patients increased from .9% in 2018 to 46.1% in 2023 (+45.1 percentage points [95% CI, 40.4–49.6]). Testing among nonpregnant patients rose from 1.2% to 35.9% (+34.6 percentage points [95% CI, 33.3–35.9]). Overall, only 1,068 of 5,178 pregnant encounters received a syphilis test (20.6%). Among 1,068 syphilis-tested pregnant encounters, 15 (1.4%) were treponemal-positive and 9 (.8%; 843 per 100,000 tested) met the Centers for Disease Control and Prevention surveillance definition for active syphilis (approximately 1 per 119 tests). Using all pregnant encounters with GC/CT testing as the denominator, the encounter-based active syphilis rate was 173.8 per 100,000 (.17%). HIV testing increased similarly (pregnant: .4% in 2018 to 44.5% in 2023), while HIV positivity remained rare (2 pregnant; 13 overall).

**Conclusion:**

Syphilis testing and case detection increased during the postimplementation period following introduction of a preselected STI order set in the electronic health record. Many encounters in the analytic cohort still did not include syphilis testing. These findings support the ED as an important setting for identifying syphilis in pregnancy while underscoring the need for further work to understand remaining testing gaps.

## INTRODUCTION

Congenital syphilis, which can cause stillbirth, infant death, and severe morbidity, has risen more than tenfold in the US between 2012 and 2023.^1^,^2^ In Ohio, case numbers rose from 22 in 2018 to 93 in 2022.^3^ Early detection and treatment are critical to prevention. Centers for Disease Control and Prevention (CDC) guidelines recommend screening at the first prenatal visit for all pregnant individuals, with repeat testing in the early third trimester and at delivery for those at elevated risk or without prior testing.^4^

Congenital syphilis disproportionately affects infants born to socially vulnerable women with limited prenatal care.^1^ In surveillance data, 41% of cases occurred after no timely maternal test, and most of these mothers had no prenatal care.^5^ In Clark County, Nevada, between 2017 and 2022 more than half of mothers delivering a child with congenital syphilis had an emergency department (ED) visit > 30 days before delivery, yet only two-thirds were tested then.^6^ Together, these findings highlight missed opportunities—and suggest the ED visit, including pregnancy confirmation, as a practical point for standardized, electronic health record (EHR)-driven syphilis screening. A positive pregnancy test in the ED is an opportunity to screen and treat early, preventing fetal loss and congenital infection.^7^–^10^

In prior work, an EHR-based order set increased HIV co-testing for ED patients undergoing gonorrhea and chlamydia (GC/CT) testing and included a preselected option for syphilis screening.^11^ Testing for GC/CT provided the cohort most relevant to this intervention. In the ED, patients undergoing GC/CT testing represent a higher-risk population in whom broader sexually transmitted infection (STI) evaluation, including syphilis and HIV testing, is often clinically appropriate and guideline supported. We therefore focused on encounters with both pregnancy and GC/CT testing because pregnancy defines a subgroup in which syphilis screening is especially important, while GC/CT testing identifies visits in which STI evaluation is already in progress and syphilis testing could be added during the same encounter.

Using that cohort, we evaluated this order set in a retrospective, quasi-experimental study across 20 EDs. Our main objective was to determine whether syphilis testing increased among ED encounters with both pregnancy and GC/CT testing, overall and by ED site. Secondary objectives were to assess changes in HIV testing and to describe syphilis and HIV detection, including active syphilis among pregnant patients, using both test-based and ED encounter–based denominators.

## METHODS

### Setting and Study Population

We conducted a retrospective quasi-experimental study using EHR data from all patients aged 15–44 years who received both pregnancy and GC/CT testing in one of 20 EDs within the Cleveland Clinic’s Northeast Ohio health system between January 1, 2018, and December 31, 2023. Syphilis and HIV co-testing were evaluated, stratified by pregnancy status. Pregnancy testing in the ED is typically ordered at clinician discretion (eg, for abdominal pain, vaginal bleeding, or prior to imaging or medication use).

**Population Health Research Capsule**

What do we already know about this issue?

*Congenital syphilis is rising, and pregnancy-related emergency department (ED) visits for sexually transmitted infection testing may miss syphilis screening*.

What was the research question?

*Did a preselected order set increase syphilis testing in pregnant gonorrhea/chlamydia-tested encounters?*

What was the major finding of the study?

*In pregnant gonorrhea/chlamydia-tested encounters, syphilis testing rose from 0.9% to 46.1%, an increase of 45.1 percentage points (95% CI, 40.4–49.6)*.

How does this improve population health?

*Electronic order sets may expand syphilis screening during pregnancy-related ED gonorrhea/chlamydia testing and identify untreated infection*.

The Cleveland Clinic Institutional Review Board reviewed this project and determined that it constitutes internal quality improvement within standard health care operations and therefore does not represent human subjects research requiring formal approval.

### Implementation Strategy

The implementation strategy consisted of an EHR-based STI order set and audit-and-feedback. Beginning in October 2019, when clinicians ordered GC/CT testing, syphilis and HIV serology were preselected by default in a standardized order set, which could be deselected only with an active choice. Implementation was supported by staff and director meetings and periodic feedback reports summarizing site- and clinician-level co-testing rates. Co-testing rates and the shift from stand-alone to bundled syphilis-plus-HIV orders served as quantitative indicators of adoption and implementation fidelity. Core elements—the preselected syphilis and HIV tests within a single order set and the audit-and-feedback reports—were specified a priori and remained unchanged across sites, while ED leaders had flexibility in how they promoted and reinforced the order set locally.^11^

We compared changes in syphilis testing over time among patients who received both pregnancy and GC/CT testing before and after implementation. Unless otherwise specified, all analyses used a consistent denominator of ED encounters in which both pregnancy and GC/CT tests were performed.

### Data Sources, Laboratory Methods, and Chart Review

Demographic, pregnancy, and STI data were retrieved from Epic Clarity (Epic Systems Corporation, Verona, WI) and stored within a secure, password-protected institutional Teradata DataLab environment. Primary measures were extracted electronically from structured EHR fields. For treponemal-positive results, one author (CBF), an infectious diseases physician, performed targeted chart review to adjudicate whether cases met the CDC 2018 surveillance case definition for active syphilis. This review was not blinded to study aims, and no formal interrater reliability assessment was performed.^12^

To screen for syphilis, we used a reverse-sequence testing algorithm: treponemal antibody screen followed by rapid plasma reagin (RPR) test where the former was reactive. If the RPR was nonreactive, a second treponemal antibody assay was added to adjudicate discordant results. The initial treponemal antibody screen was performed using Bio-Plex 2200 Syphilis IgG test (Bio-Rad Laboratories Inc, Hercules, CA) through September 2019, followed by the Bio-Plex 2200 Syphilis total IgM/IgG test from October 2019 to February 2022, and thereafter the Syphilis TP test (Abbott Laboratories, Abbott, IL). The latter test detects both IgM and IgG antibodies. A reactive or equivocal treponemal antibody result was followed by the Macro-Vue RPR Card test kit (Becton, Dickinson and Company, Franklin Lakes, NJ). If the RPR was nonreactive, confirmation was performed with Trep-Sure enzyme-linked immunoassay (Trinity Biotech plc, Bray, County Wicklow, Ireland). All the treponemal antibody assays used highly purified recombinant antigens of *Treponema pallidum*.

Operational definitions followed CDC guidance.^13^,^14^ Results were categorized as follows: a) treponemal antibody false-positive result—a reactive initial treponemal antibody screening assay followed by a nonreactive confirmatory treponemal antibody assay; and b) confirmed treponemal-positive syphilis—a reactive screening and confirmatory treponemal antibody assay, regardless of nontreponemal test result. This category includes active infection, previously treated infection, and very recent infections prior to nontreponemal (RPR) seroconversion.

- Cases with a reactive nontreponemal test (eg, RPR) and compatible clinical or epidemiologic findings were considered active syphilis.
- Cases with both a reactive treponemal antibody test and reactive RPR, along with compatible signs and symptoms, were classified as active syphilis per CDC guidance. This distinction separated active cases from serofast individuals and those with treated syphilis but biologically false-positive RPR results (eg, due to HIV infection).
- Cases with nonreactive or low-titer (serofast) RPR results, absence of symptoms, and documentation of prior adequate therapy—with no epidemiologic or laboratory evidence of reinfection (≥ 4-fold rise in RPR titer) or treatment failure—were classified as previously treated syphilis.
  (c) Active syphilis (CDC surveillance case definition): cases meeting the CDC 2018 Surveillance Case
  Definition—typically treponemal antibody–positive with a reactive RPR and compatible clinical or epidemiologic criteria. Patients with documented prior adequate treatment and no evidence of treatment failure or reinfection were excluded from this category.

For tests performed after December 31, 2019, EHR result labels (“Active Treponemal Infection,” “Treated Past Treponemal Infection”) were mapped to these categories (including treponemal antibody false-positive results when applicable); earlier results and active-case status were adjudicated by the infectious diseases physician’s chart abstraction described above.

## OTHER SEXUALLY TRANSMITTED INFECTIONS

In addition to syphilis, test results for HIV, *Chlamydia trachomatis*, and *Neisseria gonorrhoeae* were retrieved from the EHR.

### Study Outcomes

The primary study outcome was syphilis testing during ED encounters with both pregnancy and GC/CT testing, overall and by ED site. Secondary outcomes included HIV testing, combined syphilis plus HIV co-testing, confirmed treponemal-positive syphilis, active syphilis per CDC case definition among pregnant patients, and HIV positivity, reported both as proportions among those tested and as rates per 100,000 ED encounters in the analytic cohort.

### Statistical Analysis

We summarized demographics descriptively. Testing rates and positivity were reported as proportions with percentages and 95% CIs, stratified by year and pregnancy status. We used Wilson score intervals for proportions; exact (Clopper–Pearson) intervals were applied for small or extreme values. Absolute differences were expressed as percentage-point changes with Newcombe 95% CIs. When shown, rate ratios were calculated using the Katz method with continuity correction. Rates per 100,000 were based on either the number tested or the broader cohort screened for both pregnancy and GC/CT, with CIs scaled accordingly. Age was summarized as median [IQR]. As analyses were descriptive and not hypothesis-driven, P values were not reported.

A site-level analysis compared each ED’s change in syphilis testing between 2018 and 2023 (y-axis) with its 2023 share of network testing volume (x-axis), with bubble size proportional to the absolute increase in tests performed. Median values of change in testing and volume share defined four quadrants—high-volume/high-gain; high-volume/low-gain; low-volume/high-gain; and low-volume/low-gain—to identify sites contributing most to network-wide improvement.

All eligible ED encounters during 2018–2023 were included. We did not perform a priori sample size calculation because analyses were descriptive and based on the full available cohort. Data management was performed using Statistical Analysis System 9.4 (SAS Institute, Cary, NC). Analyses were conducted using SAS and Python 3.12 (pandas, NumPy, SciPy [scipy.stats], Matplotlib). This study was reported in accordance with the Strengthening the Reporting of Observational Studies in Epidemiology (STROBE) guideline.^15^

## RESULTS

### Population Characteristics

Among 59,171 encounters in the analytic cohort, 5,178 (8.8%) were among pregnant patients. Median age was similar between pregnant and nonpregnant patients (24 versus 25 years). Pregnant patients were more likely to have Medicaid insurance, be unemployed, and have slightly greater interpreter need. Additional demographic details appear in Table 1.

### STI Testing Overall

Syphilis testing was the primary outcome and the study’s clearest finding. Positivity for GC/CT was more common than syphilis or HIV positivity in both pregnancy groups (Table 2). Overall STI and GC/CT positivity were lower among pregnant encounters.

### Syphilis Testing Over Time

Syphilis testing increased markedly in both pregnant and nonpregnant patients during the study period (Figure 1). Among pregnant patients, testing rose from 0.9% in 2018 to 46.1% in 2023, an absolute increase of 45.1 percentage points (95% CI, 40.4–49.6); among nonpregnant encounters, it rose from 1.2% to 35.9%, an increase of 34.6 percentage points (95% CI, 33.3–35.9). Over time, syphilis testing in pregnant encounters shifted from occasional stand-alone ordering toward testing more often paired with HIV testing, narrowing the difference between syphilis testing and combined syphilis/HIV co-testing.

Among the 15 patients with confirmed treponemal-positive serology, nine met CDC surveillance criteria for active syphilis (Table 2) and required treatment, including one with evidence of reinfection; the remaining six had serologic evidence of previously treated infection without reinfection or treatment failure. The active syphilis rate was 843 per 100,000 among those tested and 173.8 per 100,000 among all pregnant encounters with GC/CT testing. Treponemal false-positive results were rare in both pregnancy groups. Pregnancy-specific yearly trends in confirmed treponemal-positive and active syphilis are shown in Figure 2 and Table 3.

### Characteristics of Syphilis-Tested and Positive Patients

Syphilis-tested encounters differed from those not tested on several demographic measures, and treponemal-positive patients were more often older and socially vulnerable on measures such as Medicaid insurance and unemployment. Details appear in Supplemental Tables 1–4, which compare tested versus untested encounters overall and in the pregnant subgroup, and treponemal-positive versus negative encounters overall and in the pregnant subgroup.

### HIV Testing

Testing for HIV also increased during the study period, but HIV positivity remained rare (Tables 2–4). Among pregnant encounters, HIV testing rose from 0.4% in 2018 to 44.5% in 2023; among nonpregnant encounters it rose from 0.9% to 32.8% (Tables 3–4). Overall, 13 patients tested HIV positive, including 2 pregnant patients. No patients were co-infected with both HIV and syphilis.

### Site-Level Variation in Syphilis Testing

Improvement in syphilis testing was not uniform across EDs (Figure 3). Across all 20 EDs, the mean syphilis testing rate increased by 36 percentage points from 2018 to 2023, but a subset of high-volume sites accounted for most of that gain. Seven high-volume/high-gain EDs represented 72% of 2023 encounters and 85% of the absolute increase in testing, whereas three additional high-volume sites accounted for 11% of encounters but only 7% of added tests. Similar heterogeneity was present in the pregnant subgroup (Supplemental Figure 1): the mean testing rate increased by 45 percentage points, and 8 high-volume/high-gain EDs, representing 78% of 2023 volume, accounted for 88% of added tests.

## DISCUSSION

The central finding of this study was a substantial increase in syphilis testing following introduction of a preselected EHR STI order set. The pattern of improvement, including marked variation across EDs, suggests that the order set contributed to broader testing. That broader testing identified additional infections, including among pregnant patients, and revealed a syphilis burden among ED patients evaluated for urogenital STIs that exceeded rates reported for the general population of women aged 15–44 years. Among syphilis-tested pregnant encounters, we identified approximately one active case per 119 tests, underscoring both the feasibility and the clinical importance of ED-based detection during pregnancy.

The ED often serves as a safety-net setting for individuals with limited access to routine prenatal or primary care. In many EDs, however, STI evaluation is more likely to include gonorrhea and chlamydia testing than serologic screening for infections such as syphilis or HIV. In that context, a bundled order set may help normalize more complete STI evaluation within an existing clinical workflow.

Our findings extend prior work on ED STI order sets in two ways: by focusing on syphilis testing during the postimplementation period across 20 EDs; and by describing clinically meaningful detection outcomes, particularly active syphilis among pregnant patients. Similar interventions have increased syphilis screening in other ED settings, including among pregnant patients, and our findings are consistent with that literature.^8^,^16^ In an adult Level I trauma ED in Chicago, an opt-out workflow built on existing HIV screening infrastructure increased syphilis screening substantially, with the largest gains among pregnant patients and a marked rise in diagnoses.^17^ Data from Clark County, NV, further underscore the importance of the ED setting: Many mothers of infants with congenital syphilis had an ED visit before delivery but were not tested for syphilis at that visit.^6^ In our cohort, testing before implementation was rare among encounters with both pregnancy and GC/CT testing. During the postimplementation period, testing rose substantially, and active syphilis requiring treatment was identified among pregnant patients.

The active syphilis rate among pregnant patients in our ED cohort was 843 per 100,000 among those tested for syphilis and 173.8 per 100,000 among all pregnant encounters with GC/CT testing. These rates are notable in the context of Cuyahoga County, where our health system is based and where the reported syphilis rate among women aged 15–44 years is 22.7 per 100,000—already well above the CDC threshold of 4.6 per 100,000 used to define areas for intensified screening.^18^,^19^ According to CDC guidelines, all pregnant persons should be screened for syphilis at the first prenatal visit, with repeat testing in the early third trimester and at delivery for those at increased risk or in areas of high prevalence. The American College of Obstetricians and Gynecologists recommends universal screening at all three time points.^20^ Despite these recommendations, populations at highest risk for syphilis often have inadequate or no prenatal care, making the ED their only opportunity for early detection.^6^

Although syphilis testing increased substantially during the post-implementation period, many encounters in the analytic cohort still did not include syphilis testing. Implementation was not uniform across EDs. Low ED syphilis co-testing has also been reported nationally among patients undergoing GC/chlamydia testing, suggesting that low baseline co-testing is common in this clinical context. In a national ED sample, syphilis testing was performed in only 2.9% of GC/chlamydia encounters, and combined HIV-syphilis cotesting in 1.5%.^21^ In our system, testing increased substantially over time, but that increase was concentrated in a subset of EDs, which likely contributed to the lower overall testing proportion across the full cohort. Among pregnant encounters, syphilis testing also became increasingly paired with HIV testing over time, suggesting that the bundled workflow was becoming more consistently incorporated into routine practice. Because we did not directly measure clinician-, workflow-, or patient-level reasons for nontesting, we cannot determine why testing remained incomplete at specific encounters. These findings therefore identify a persistent implementation gap.

Interpretation of these findings requires caution. This was a retrospective before-after study conducted during a period of broader change in ED utilization and STI testing practice, including the COVID-19 pandemic. Accordingly, the observed increase in testing during the postimplementation period should be interpreted as a temporal association rather than as proof that the order set alone caused the increase. The uneven pattern of improvement across EDs nevertheless suggests that local implementation and reinforcement contributed to the observed differences in performance, rather than the increase reflecting only a uniform secular trend across the health system.

The intervention was also simple to deploy. We did not perform a formal economic evaluation, but implementation required approximately 6 hours of EHR analyst time for build, testing, and deployment (≈$450 total), with about 2 hours of annual maintenance. Result follow-up used an existing ED pharmacy callback workflow.

## LIMITATIONS

This study has several limitations. First, we did not perform adjusted analyses to isolate the independent association of the order set with testing outcomes. Accordingly, the observed increase in testing may reflect a combination of implementation strategy, changes in patient mix across years, site-level practice variations, and broader secular trends. This limitation is particularly important because the order set was introduced in October 2019, only a few months before the COVID-19 pandemic disrupted ED utilization, staffing, workflow, and STI testing practices. The observed increases should therefore be interpreted as temporal associations during the postimplementation period rather than as evidence that the order set alone caused the increase.

Second, we did not perform chart reviews in nonpregnant patients to determine whether confirmed treponemal-positive serologic results met the CDC case definition for syphilis or reflected previously treated infection.^13^ Consequently, the confirmed treponemal-positive rate should not be interpreted as the incidence of active syphilis, as it includes both current and previously treated cases. Third, treatment completion and clinical outcomes were not assessed. Although positive results were routed through an existing ED pharmacist callback workflow and reported to the Department of Health, treatment completion was not measured. Fourth, the study period included changes in treponemal assays, with the replacement of a syphilis IgG assay by syphilis total IgG/IgM assays with a narrower window period. However, all assays were used within an accepted reverse-sequence testing framework, and we do not believe these changes materially altered the study’s main descriptive findings. Fifth, we did not measure whether the intervention reduced congenital syphilis incidence. Finally, as with any screening program, false-positive results and follow-up burden remain important considerations, although treponemal false-positive results were uncommon in our cohort.^22^–^26^

## CONCLUSION

Syphilis testing increased substantially during the postimplementation period following introduction of a preselected EHR order set. Although detection in pregnancy was clinically meaningful, testing remained incomplete across the analytic cohort. These findings support the ED as an important setting for screening while highlighting persistent variation in implementation across sites.

## Supplementary Information



## Figures and Tables

**Figure 1:**
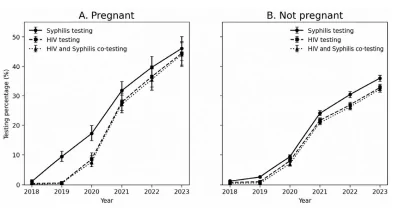
Syphilis and HIV testing over time among emergency department patients tested for both pregnancy and gonorrhea/chlamydia, 2018–2023. Panel A: Annual testing rates (%) among pregnant patients tested for both pregnancy and gonorrhea/chlamydia. Panel B: Corresponding rates (%) among nonpregnant patients tested for both pregnancy and gonorrhea/chlamydia. Each panel displays annual rates of syphilis testing, HIV testing, and combined HIV + syphilis co-testing. Error bars represent 95% confidence intervals. The EHR-based order set promoting HIV and syphilis co-testing was implemented in October 2019. In a retrospective, quasi-experimental EHR study across 20 EDs (2018–2023) of encounters among patients aged 15–44 years who received both pregnancy and GC/CT testing. *HIV*, human immunodeficiency virus; *ED*, emergency department; *EHR*, electronic health record; *GC/CT*, gonorrhea/chlamydia.

**Figure 2:**
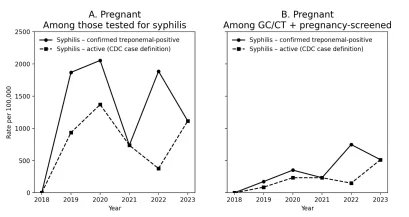
Syphilis detection among pregnant patients tested in the emergency department for both pregnancy and gonorrhea/chlamydia, 2018–2023. Panel A: Annual rates (per 100,000) among pregnant patients tested for syphilis. Panel B: Corresponding rates (per 100,000) among all pregnant patients tested for both pregnancy and gonorrhea/chlamydia (GC/CT), regardless of whether syphilis testing was performed. Each panel displays confirmed treponemal-positive encounters and the subset meeting the CDC surveillance criterion for active syphilis. Confidence intervals are omitted for visual clarity; exact values appear in Table 3. *CDC*, Centers for Disease Control and Prevention.

**Figure 3:**
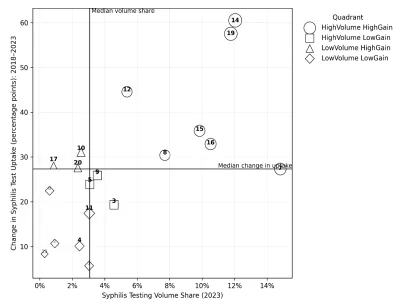
Site-level variation in syphilis testing among emergency department patients tested for both pregnancy and gonorrhea/chlamydia, 2018–2023. This plot compares each ED’s change in syphilis testing from 2018 to 2023 (y-axis) with its 2023 share of total network testing volume (x-axis). Each point represents one ED; bubble size is proportional to the absolute increase in tests performed. Median volume share and median testing change define four quadrants (high-volume/high-gain; high-volume/low-gain; low-volume/high-gain; low-volume/low-gain), illustrating that most network-wide improvement was concentrated among a few large, high-performing EDs.

**Table 1:** Demographic characteristics of encounters among patients tested for pregnancy and gonorrhea/chlamydia in a study to evaluate whether a standardized order set would increase syphilis testing.

Factor	Not pregnant	Pregnant
Denominator (group totals)	*n* = 53,993	*n* = 5,178
Age at encounter	25.0 [21.0, 31.0]	24.0 [21.0, 29.0]
Homeless		
Yes	174 (0.3% [0.3–0.4])	13 (0.3% [0.1–0.4])
Interpreter Needed		
Yes	791 (1.5% [1.4–1.6])	119 (2.3% [1.9–2.7])
Race/Ethnicity		
Hispanic	5,058 (9.4% [9.1–9.6])	575 (11.1% [10.3–12.0])
Non-Hispanic White	12,818 (23.7% [23.4–24.1])	932 (18.0% [17.0–19.1])
Non-Hispanic Black	33,545 (62.1% [61.7–62.5])	3,405 (65.8% [64.5–67.0])
Non-Hispanic Asian	260 (0.5% [0.4–0.5])	24 (0.5% [0.3–0.7])
Other/Declined	2,312 (4.3% [4.1–4.5])	242 (4.7% [4.1–5.3])
Employment		
Full-time/Retired	19,280 (35.7% [35.3–36.1])	1,698 (32.8% [31.5–34.1])
Part-time	4,433 (8.2% [8.0–8.4])	406 (7.8% [7.1–8.6])
Unemployed	29,061 (53.8% [53.4–54.2])	2,974 (57.4% [56.1–58.8])
Student	665 (1.2% [1.1–1.3])	36 (0.7% [0.5–1.0])
Unknown	554 (1.0% [0.9–1.1])	64 (1.2% [1.0–1.6])
Language		
English	52,546 (97.3% [97.2–97.5])	4,973 (96.0% [95.5–96.5])
Spanish	1,035 (1.9% [1.8–2.0])	139 (2.7% [2.3–3.2])
Other	412 (0.8% [0.7–0.8])	66 (1.3% [1.0–1.6])
Marital Status		
Single	46,560 (86.2% [85.9–86.5])	4,467 (86.3% [85.3–87.2])
Married/Partner	5,537 (10.3% [10.0–10.5])	601 (11.6% [10.8–12.5])
Divorced/Separated	1487 (2.8% [2.6–2.9])	90 (1.7% [1.4–2.1])
Other	112 (0.2% [0.2–0.2])	6 (0.1% [0.1–0.3])
Unknown	297 (0.6% [0.5–0.6])	14 (0.3% [0.2–0.5])
Primary Payor		
Selfpay	5,295 (9.8% [9.6–10.1])	492 (9.5% [8.7–10.3])
Private	10,303 (19.1% [18.8–19.4])	804 (15.5% [14.6–16.5])
Medicare	1,088 (2.0% [1.9–2.1])	70 (1.4% [1.1–1.7])
Medicaid	37,062 (68.6% [68.2–69.0])	3,797 (73.3% [72.1–74.5])
TRICARE/Military	169 (0.3% [0.3–0.4])	9 (0.2% [0.1–0.3])
International	11 (0.0% [0.0–0.0])	0 (0.0% [0.0–0.1])
Uninsured	65 (0.1% [0.1–0.2])	6 (0.1% [0.1–0.3])

Continuous variables are reported as median [P25, P75].

*ED*, emergency department; *EHR*, electronic health record.

**Table 2:** Sexually transmitted infection co-testing and results among ED patients aged 15–44 years tested for both pregnancy and gonorrhea/chlamydia.

Factor	Not pregnant	Pregnant	Difference (Pregnant - Not pregnant)
STI Testing Among GC/CT-Screened Encounters			
Syphilis test	8,470/53,993 (15.7% [15.4–16.0])	1,068/5178 (20.6% [19.5–21.7])	4.9 pp [3.5–6.4]
HIV test	7,440/53,993 (13.8% [13.5–14.1])	826/5178 (16.0% [15.0–17.0])	2.2 pp [0.9–3.5]
HIV and Syphilis co-testing	7,090/53,993 (13.1% [12.8–13.4])	799/5178 (15.4% [14.5–16.4])	2.3 pp [1.1–3.6]
STI Positivity Among Those Tested			
≥1 positive result	6,653/53,993 (12.3% [12.0–12.6])	511/5,178 (9.9% [9.1–10.7])	–2.5 pp [–3.5–−1.3]
GC/CT positive	6,491/53,993 (12.0% [11.8–12.3])	494/5,178 (9.5% [8.8–10.4])	–2.5 pp [–3.5–−1.4]
Confirmed treponemal-positive syphilis	184/8,470 (2.2% [1.9–2.5])	15/1068 (1.4% [0.9–2.3])	–0.8 pp [−1.7–0.4]
Active syphilis (CDC surveillance case definition)	—	9/1068 (0.8% [0.4–1.6])	—
Syphilis – false-positive	32/8,470 (0.4% [0.3–0.5])	5/1068 (0.5% [0.2–1.1])	0.1 pp [–0.3–0.8]
HIV positive	11/7,440 (0.1% [0.1–0.3])	2/826 (0.2% [0.1–0.9])	0.1 pp [–0.2–0.8]
HIV and Syphilis both positive	0/7090 (0.0% [0.0–0.1])	0/799 (0.0% [0.0–0.5])	0.0 pp [–0.1–0.5]
Positivity per 100,000 Among GC/CT-Screened Encounters			
Confirmed treponemal-positive syphilis	184 (340.8 per 100,000 [295.0–393.6])	15 (289.7 per 100,000 [175.6–477.4])	—
Active syphilis (CDC surveillance case definition)	—	9 (173.8 per 100,000 [91.5–330.0])	—
HIV Positive	11 (20.4 per 100,000 [11.4–36.5])	2 (38.6 per 100,000 [10.6–140.7])	—

Testing rows use the GC/CT-screened cohort as the denominator and are reported as n/N (% [95% CI]). Positivity rows are reported among those tested for the specified assay. Per-100,000 rows use the GC/CT-screened cohort as the denominator. Active syphilis reflects the subset of confirmed treponemal-positive pregnant encounters meeting CDC surveillance criteria.

C*DC*, Centers for Disease Control and Prevention; *ED*, emergency department; *GC/CT*, gonorrhea/chlamydia; *pp*, percentage points.

**Table 3:** Sexually transmitted infection testing trends and results for pregnant patients in the emergency department tested for both pregnancy and gonorrhea/chlamydia.

Factor	2018	2019	2020	2021	2022	2023	Change 2018→2023	Ratio 2023/2018
Yearly Trends in STI Testing Among *GC/CT*Screened Encounters								
Denominator per year	*N* = 1,074	*N* = 1,144	*N* = 853	*N* = 855	*N* = 668	*N* = 584		
Syphilis test	10 (0.9% [0.5–1.7])	107 (9.4% [7.8–11.2])	146 (17.1% [14.7–19.8])	271 (31.7% [28.7–34.9])	265 (39.7% [36.0–43.4])	269 (46.1% [42.1–50.1])	45.1 pp [40.4–49.6]	49.47 [24.66–98.94]
HIV test	4 (0.4% [0.1–1.0])	7 (0.6% [0.3–1.3])	73 (8.6% [6.9–10.6])	238 (27.8% [24.9–30.9])	244 (36.5% [33.0–40.2])	260 (44.5% [40.5–48.6])	44.1 pp [39.6–48.4]	119.54 [42.51–335.16]
HIV and Syphilis co-testing	2 (0.2% [0.1–0.7])	6 (0.5% [0.2–1.1])	65 (7.6% [6.0–9.6])	232 (27.1% [24.3–30.2])	237 (35.5% [31.9–39.2])	257 (44.0% [40.0–48.1])	43.8 pp [39.4–48.0]	236.32 [59.18–940.80]
Yearly STI Positivity Rate Among Those Tested								
≥1 positive result	90/1074 (8.4% [6.9–10.2])	101/1144 (8.8% [7.3–10.6])	79/853 (9.3% [7.5–11.4])	106/855 (12.4% [10.4–14.8])	72/668 (10.8% [8.6–13.4])	63/584 (10.8% [8.5–13.6])	2.4 pp [−1.7–6.7]	1.29 [0.84–1.98]
GC/CT positive	90/1074 (8.4% [6.9–10.2])	99/1144 (8.7% [7.2–10.4])	76/853 (8.9% [7.2–11.0])	103/855 (12.0% [10.0–14.4])	66/668 (9.9% [7.8–12.4])	60/584 (10.3% [8.1–13.0])	1.9 pp [−2.1–6.1]	1.23 [0.79–1.89]
Confirmed treponemal-positive syphilis	0/10 (0.0% [0.0–30.8])	2/107 (1.9% [0.5–6.6])	3/146 (2.1% [0.7–5.9])	2/271 (0.7% [0.2–2.7])	5/265 (1.9% [0.8–4.3])	3/269 (1.1% [0.4–3.2])	1.1 pp [−27.4–3.2]	—
Active syphilis (CDC surveillance case definition)	0/10 (0.0% [0.0–30.8])	1/107 (0.9% [0.2–5.1])	2/146 (1.4% [0.4–4.9])	2/271 (0.7% [0.2–2.7])	1/265 (0.4% [0.1–2.1])	3/269 (1.1% [0.4–3.2])	1.1 pp [–27.4–3.2]	—
Syphilis – false-positive	1/10 (10.0% [1.8–40.4])	0/107 (0.0% [0.0–3.4])	1/146 (0.7% [0.1–3.8])	2/271 (0.7% [0.2–2.7])	1/265 (0.4% [0.1–2.1])	0/269 (0.0% [0.0–1.4])	–10.0 pp [–40.4–−0.4]	0.00 [0.00–0.79]
HIV positive	0/4 (0.0% [0.0–60.2])	0/7 (0.0% [0.0–41.0])	0/73 (0.0% [0.0–4.9])	1/238 (0.4% [0.1–2.3])	1/244 (0.4% [0.1–2.3])	0/260 (0.0% [0.0–1.4])	0.0 pp [–49.0–1.5]	—
HIV positive and confirmed treponemal-positive syphilis	0/2 (0.0% [0.0–84.2])	0/6 (0.0% [0.0–45.9])	0/65 (0.0% [0.0–5.5])	0/232 (0.0% [0.0–1.6])	0/237 (0.0% [0.0–1.5])	0/257 (0.0% [0.0–1.4])	0.0 pp [–65.8–1.5]	—
Yearly Syphilis and HIV Rates per 100,000 Among *GC/CT*Screened Encounters								
Denominator per year	N=1074	N=1144	N=853	N=855	N=668	N=584		
Confirmed treponemal-positive syphilis	0 (0.0 per 100,000 [0.0–342.9])	2 (174.8 per 100,000 [48.0–635.2])	3 (351.7 per 100,000 [119.7–1028.9])	2 (233.9 per 100,000 [64.2–848.9])	5 (748.5 per 100,000 [320.1–1740.1])	3 (513.7 per 100,000 [174.9–1499.3])		
Active syphilis (CDC surveillance case definition)	0 (0.0 per 100,000 [0.0–342.9])	1 (87.4 per 100,000 [15.4–493.5])	2 (234.5 per 100,000 [64.3–850.8])	2 (233.9 per 100,000 [64.2–848.9])	1 (149.7 per 100,000 [26.4–843.0])	3 (513.7 per 100,000 [174.9–1499.3])		
HIV positive	0 (0.0 per 100,000 [0.0–342.9])	0 (0.0 per 100,000 [0.0–321.9])	0 (0.0 per 100,000 [0.0–431.5])	1 (117.0 per 100,000 [20.6–659.5])	1 (149.7 per 100,000 [26.4–843.0])	0 (0.0 per 100,000 [0.0–629.7])		

Note: Testing rows use the GC/CT-screened cohort as the denominator (per-year denominators shown). Positivity rows are reported as n/N (% [95% CI]) among those tested.

*CDC*, Centers for Disease Control and Prevention; *GC/CT*, gonorrhea/chlamydia; *pp*, percentage points; *STI*, sexually transmitted infection.

**Table 4:** Sexually transmitted infection testing trends and results for nonpregnant ED patients aged 15–44 years tested for both pregnancy and gonorrhea/chlamydia in the emergency department.

Factor	2018	2019	2020	2021	2022	2023	Change 2018→2023	Ratio 2023/2018
Yearly Trends in STI Testing Among *GC/CT*‐Screened Encounters								
Denominator per year	*N* = 10,062	*N* = 10,815	*N* = 8,695	*N* = 8,890	*N* = 8,015	*N* = 7,516		
Syphilis test	124 (1.2% [1.0–1.5])	279 (2.6% [2.3–2.9])	813 (9.4% [8.8–10.0])	2,130 (24.0% [23.1–24.9])	2,429 (30.3% [29.3–31.3])	2,695 (35.9% [34.8–36.9])	34.6 pp [33.3–35.9]	29.10 [23.70–35.71]
HIV test	87 (0.9% [0.7–1.1])	114 (1.1% [0.9–1.3])	695 (8.0% [7.4–8.6])	1,930 (21.7% [20.9–22.6])	2,148 (26.8% [25.8–27.8])	2,466 (32.8% [31.8–33.9])	31.9 pp [30.7–33.2]	37.95 [29.81–48.29]
HIV and Syphilis co-testing	52 (0.5% [0.4–0.7])	63 (0.6% [0.5–0.7])	600 (6.9% [6.4–7.5])	1,864 (21.0% [20.1–21.8])	2,088 (26.1% [25.1–27.0])	2,423 (32.2% [31.2–33.3])	31.7 pp [30.5–32.9]	62.38 [46.07–84.45]
Yearly STI Positivity Rate Among Those Tested								
≥1 positive result	957/10,062 (9.5% [9.0–10.1])	1,134/10,815 (10.5% [9.9–11.1])	1,265/8,695 (14.5% [13.8–15.3])	1,326/8,890 (14.9% [14.2–15.7])	1,049/8,015 (13.1% [12.4–13.8])	922/7,516 (12.3% [11.5–13.0])	2.8 pp [1.4–4.1]	1.29 [1.14–1.46]
GC/CT positive	953/10,062 (9.5% [8.9–10.1])	1,130/10,815 (10.4% [9.9–11.0])	1,257/8,695 (14.5% [13.7–15.2])	1,294/8,890 (14.6% [13.8–15.3])	1,006/8,015 (12.6% [11.8–13.3])	851/7,516 (11.3% [10.6–12.1])	1.9 pp [0.6–3.1]	1.20 [1.06–1.35]
Confirmed treponemal-positive syphilis	3/124 (2.4% [0.8–6.9])	4/279 (1.4% [0.6–3.6])	11/813 (1.4% [0.8–2.4])	37/2,130 (1.7% [1.3–2.4])	55/2,429 (2.3% [1.7–2.9])	74/2,695 (2.7% [2.2–3.4])	0.3 pp [–4.7–2.6]	1.13 [0.32–4.16]
Syphilis – false-positive	2/124 (1.6% [0.4–5.7])	0/279 (0.0% [0.0–1.3])	8/813 (1.0% [0.5–1.9])	9/2,130 (0.4% [0.2–0.8])	6/2,429 (0.2% [0.1–0.5])	7/2,695 (0.3% [0.1–0.5])	–1.4 pp [–5.6–0.1]	0.16 [0.02–1.21]
HIV positive	2/87 (2.3% [0.6–8.0])	1/114 (0.9% [0.2–4.8])	0/695 (0.0% [0.0–0.5])	1/1,930 (0.1% [0.0–0.3])	1/2,148 (0.0% [0.0–0.3])	6/2,466 (0.2% [0.1–0.5])	–2.1 pp [–7.9–−0.1]	0.11 [0.01–0.84]
HIV positive and confirmed treponemal-positive syphilis	0/52 (0.0% [0.0–6.8])	0/63 (0.0% [0.0–5.7])	0/600 (0.0% [0.0–0.6])	0/1,864 (0.0% [0.0–0.2])	0/2,088 (0.0% [0.0–0.2])	0/2,423 (0.0% [0.0–0.2])	0.0 pp [–6.9–0.2]	—
Yearly Syphilis and HIV Rates per 100,000 Among *GC/CT*‐Screened Encounters								
Denominator per year	*N* = 10,062	*N* = 10,815	*N* = 8,695	*N* = 8,890	*N* = 8,015	*N* = 7,516		
Confirmed treponemal-positive syphilis	3 (29.8 per 100,000 [10.1–87.6])	4 (37.0 per 100,000 [14.4–95.1])	11 (126.5 per 100,000 [70.7–226.4])	37 (416.2 per 100,000 [302.1–573.1])	55 (686.2 per 100,000 [527.6–892.1])	74 (984.6 per 100,000 [785.0–1234.2])		
HIV positive	2 (19.9 per 100,000 [5.5–72.5])	1 (9.2 per 100,000 [1.6–52.4])	0 (0.0 per 100,000 [0.0–42.4])	1 (11.2 per 100,000 [2.0–63.7])	1 (12.5 per 100,000 [2.2–70.6])	6 (79.8 per 100,000 [36.6–174.1])		

Testing rows use the gonorrhea/chlamydia-screened cohort as the denominator (per-year denominators shown). Positivity rows are reported as n/N (% [95% CI]) among those tested. Per-100,000 rows use the gonorrhea/chlamydia-screened cohort as the denominator.

*GC/CT*, gonorrhea/chlamydia; *pp*, percentage points.
