# Supplementary material for: Syphilis Screening in Pregnancy During Emergency Department Gonorrhea and Chlamydia Testing
Source: West J Emerg Med. 2026 Sep 24;27(5):1467–77. doi: 10.5811/westjem.54849 (PMC13660586; doi:10.5811/westjem.54849)
Supplement: Supplementary file 1 [file wjem-27-1467-s001.docx]

**Supplemental Table 1.** Demographic characteristics of all ED patients with and without syphilis co-testing

| Factor | Not co‑tested N=49633 | Co‑tested N=9538 | Difference (Co‑tested - Not co‑tested) |
| --- | --- | --- | --- |
| Age At Encounter | 25.0 [21.0, 31.0] | 25.0 [21.0, 31.0] |  |
| Homeless |  |  |  |
| Yes | 125 (0.3% [0.2–0.3]) | 62 (0.7% [0.5–0.8]) | 0.4 pp [0.2–0.6] |
| Interpreter Needed |  |  |  |
| Yes | 726 (1.5% [1.4–1.6]) | 184 (1.9% [1.7–2.2]) | 0.5 pp [0.1–0.9] |
| Race/Ethnicity |  |  |  |
| Hispanic | 4456 (9.0% [8.7–9.2]) | 1177 (12.3% [11.7–13.0]) | 3.4 pp [2.5–4.3] |
| Non‑Hispanic White | 11939 (24.1% [23.7–24.4]) | 1811 (19.0% [18.2–19.8]) | -5.1 pp [-6.2–-3.9] |
| Non‑Hispanic Black | 30854 (62.2% [61.7–62.6]) | 6096 (63.9% [62.9–64.9]) | 1.7 pp [0.4–3.1] |
| Non‑Hispanic Asian | 251 (0.5% [0.4–0.6]) | 33 (0.3% [0.2–0.5]) | -0.2 pp [-0.3–0.0] |
| Other/Declined | 2133 (4.3% [4.1–4.5]) | 421 (4.4% [4.0–4.8]) | 0.1 pp [-0.5–0.7] |
| Employment |  |  |  |
| Full-time/Retired | 17837 (35.9% [35.5–36.4]) | 3141 (32.9% [32.0–33.9]) | -3.0 pp [-4.4–-1.6] |
| Part-time | 4153 (8.4% [8.1–8.6]) | 686 (7.2% [6.7–7.7]) | -1.2 pp [-1.9–-0.4] |
| Unemployed | 26528 (53.4% [53.0–53.9]) | 5507 (57.7% [56.7–58.7]) | 4.3 pp [2.9–5.7] |
| Student | 599 (1.2% [1.1–1.3]) | 102 (1.1% [0.9–1.3]) | -0.1 pp [-0.4–0.2] |
| Unknown | 516 (1.0% [1.0–1.1]) | 102 (1.1% [0.9–1.3]) | 0.0 pp [-0.3–0.3] |
| Language |  |  |  |
| English | 48321 (97.4% [97.2–97.5]) | 9198 (96.4% [96.0–96.8]) | -0.9 pp [-1.4–-0.4] |
| Spanish | 903 (1.8% [1.7–1.9]) | 271 (2.8% [2.5–3.2]) | 1.0 pp [0.6–1.5] |
| Other | 409 (0.8% [0.7–0.9]) | 69 (0.7% [0.6–0.9]) | -0.1 pp [-0.3–0.2] |
| Marital Status |  |  |  |
| Single | 42491 (85.6% [85.3–85.9]) | 8536 (89.5% [88.9–90.1]) | 3.9 pp [2.9–4.8] |
| Married/Partner | 5431 (10.9% [10.7–11.2]) | 707 (7.4% [6.9–8.0]) | -3.5 pp [-4.3–-2.7] |
| Divorced/Separated | 1347 (2.7% [2.6–2.9]) | 230 (2.4% [2.1–2.7]) | -0.3 pp [-0.7–0.2] |
| Other | 100 (0.2% [0.2–0.2]) | 18 (0.2% [0.1–0.3]) | -0.0 pp [-0.1–0.1] |
| Unknown | 264 (0.5% [0.5–0.6]) | 47 (0.5% [0.4–0.7]) | -0.0 pp [-0.2–0.2] |
| Primary Payer |  |  |  |
| Self‑Pay | 5019 (10.1% [9.9–10.4]) | 768 (8.1% [7.5–8.6]) | -2.1 pp [-2.9–-1.2] |
| Private | 9684 (19.5% [19.2–19.9]) | 1423 (14.9% [14.2–15.6]) | -4.6 pp [-5.6–-3.5] |
| Medicare | 956 (1.9% [1.8–2.1]) | 202 (2.1% [1.8–2.4]) | 0.2 pp [-0.2–0.6] |
| Medicaid | 33742 (68.0% [67.6–68.4]) | 7117 (74.6% [73.7–75.5]) | 6.6 pp [5.3–7.9] |
| TRICARE/Military | 153 (0.3% [0.3–0.4]) | 25 (0.3% [0.2–0.4]) | -0.0 pp [-0.2–0.1] |
| International | 10 (0.0% [0.0–0.0]) | 1 (0.0% [0.0–0.1]) | -0.0 pp [-0.0–0.0] |
| Uninsured | 69 (0.1% [0.1–0.2]) | 2 (0.0% [0.0–0.1]) | -0.1 pp [-0.2–-0.0] |

Note: Proportions are reported as n/N (percent [95% CI]). Absolute differences are reported in percentage points (pp) with 95% CIs.

In a retrospective, quasi-experimental EHR study across 20 EDs (2018–2023) of encounters among patients aged 15–44 years who received both pregnancy and GC/CT testing.

*ED*, emergency department; *pp*, percentage points; *CI*, confidence interval; *EHR*, electronic health record; *GC/CT*, gonorrhea and chlamydia

**Supplemental Table 2.** Demographic characteristics of pregnant ED patients with and without syphilis co-testing

| Factor | Not co‑tested | Co‑tested | Difference (Co‑tested - Not co‑tested) |
| --- | --- | --- | --- |
| Denominator (group totals) | N=4110 | N=1068 |  |
| Age At Encounter | 24.0 [21.0, 29.0] | 25.0 [21.0, 29.0] |  |
| Homeless |  |  |  |
| Yes | 8 (0.2% [0.1, 0.4]) | 5 (0.5% [0.2, 1.1]) | 0.3 pp [-0.4, 0.6] |
| Interpreter Needed |  |  |  |
| Yes | 81 (2.0% [1.6, 2.4]) | 38 (3.6% [2.6, 4.8]) | 1.6 pp [0.2, 2.7] |
| Race/Ethnicity |  |  |  |
| Hispanic | 417 (10.1% [9.3, 11.1]) | 158 (14.8% [12.8, 17.0]) | 4.6 pp [2.2, 6.9] |
| Non‑Hispanic White | 771 (18.8% [17.6, 20.0]) | 161 (15.1% [13.1, 17.3]) | -3.7 pp [-6.2, -1.3] |
| Non‑Hispanic Black | 2713 (66.0% [64.5, 67.4]) | 692 (64.8% [61.9, 67.6]) | -1.2 pp [-4.4, 2.0] |
| Non‑Hispanic Asian | 21 (0.5% [0.3, 0.8]) | 3 (0.3% [0.1, 0.8]) | -0.2 pp [-0.8, 0.1] |
| Other/Declined | 188 (4.6% [4.0, 5.3]) | 54 (5.1% [3.9, 6.5]) | 0.5 pp [-1.1, 1.8] |
| Employment |  |  |  |
| Full-time/Retired | 1352 (32.9% [31.5, 34.3]) | 346 (32.4% [29.7, 35.3]) | -0.5 pp [-3.7, 2.6] |
| Part-time | 343 (8.3% [7.5, 9.2]) | 63 (5.9% [4.6, 7.5]) | -2.4 pp [-4.2, -0.9] |
| Unemployed | 2338 (56.9% [55.4, 58.4]) | 636 (59.6% [56.6, 62.5]) | 2.7 pp [-0.6, 6.0] |
| Student | 27 (0.7% [0.5, 1.0]) | 9 (0.8% [0.4, 1.6]) | 0.2 pp [-0.6, 0.7] |
| Unknown | 50 (1.2% [0.9, 1.6]) | 14 (1.3% [0.8, 2.2]) | 0.1 pp [-0.8, 0.7] |
| Language |  |  |  |
| English | 3967 (96.5% [95.9, 97.0]) | 1006 (94.2% [92.6, 95.4]) | -2.3 pp [-3.7, -0.7] |
| Spanish | 90 (2.2% [1.8, 2.7]) | 49 (4.6% [3.5, 6.0]) | 2.4 pp [0.9, 3.6] |
| Other | 53 (1.3% [1.0, 1.7]) | 13 (1.2% [0.7, 2.1]) | -0.1 pp [-1.0, 0.6] |
| Marital Status |  |  |  |
| Single | 3533 (86.0% [84.9, 87.0]) | 934 (87.5% [85.3, 89.3]) | 1.5 pp [-0.7, 3.9] |
| Married/Partner | 496 (12.1% [11.1, 13.1]) | 105 (9.8% [8.2, 11.8]) | -2.2 pp [-4.4, -0.3] |
| Divorced/Separated | 68 (1.7% [1.3, 2.1]) | 22 (2.1% [1.4, 3.1]) | 0.4 pp [-0.7, 1.2] |
| Other | 2 (0.0% [0.0, 0.2]) | 4 (0.4% [0.1, 1.0]) | 0.3 pp [-0.3, 0.6] |
| Unknown | 11 (0.3% [0.1, 0.5]) | 3 (0.3% [0.1, 0.8]) | 0.0 pp [-0.5, 0.3] |
| Primary Payer |  |  |  |
| Self‑Pay | 408 (9.9% [9.0, 10.9]) | 84 (7.9% [6.4, 9.6]) | -2.1 pp [-4.0, -0.3] |
| Private | 657 (16.0% [14.9, 17.1]) | 147 (13.8% [11.8, 16.0]) | -2.2 pp [-4.7, 0.0] |
| Medicare | 62 (1.5% [1.2, 1.9]) | 8 (0.7% [0.4, 1.5]) | -0.8 pp [-1.6, -0.2] |
| Medicaid | 2970 (72.3% [70.9, 73.6]) | 827 (77.4% [74.8, 79.8]) | 5.2 pp [2.4, 8.1] |
| TRICARE/Military | 7 (0.2% [0.1, 0.4]) | 2 (0.2% [0.1, 0.7]) | 0.0 pp [-0.5, 0.2] |
| Uninsured | 6 (0.1% [0.1, 0.3]) | 0 (0.0% [0.0, 0.4]) | -0.1 pp [-0.5, 0.0] |

Note: Proportions are reported as n/N (percent [95% CI]). Absolute differences are reported in percentage points (pp) with 95% CIs.

In a retrospective, quasi-experimental EHR study across 20 EDs (2018–2023) of encounters among patients aged 15–44 years who received both pregnancy and GC/CT testing.

*ED*, emergency department; *pp*, percentage points; *CI*, confidence interval; *EHR*, electronic health record; *GC/CT*, gonorrhea and chlamydia

**Supplemental Table 3.** Demographic characteristics of all ED patients with and without confirmed treponemal-positive syphilis serology

| Factor | Negative | Confirmed treponemal-positive | Difference (Confirmed treponemal-positive - Negative) |
| --- | --- | --- | --- |
| Denominator (group totals) | N=58972 | N=199 |  |
| Age At Encounter | 25.0 [21.0, 31.0] | 30.0 [24.0, 35.0] |  |
| Homeless |  |  |  |
| Yes | 182 (0.3% [0.3–0.4]) | 5 (2.5% [1.1–5.7]) | 2.2 pp [0.7–5.5] |
| Interpreter Needed |  |  |  |
| Yes | 909 (1.5% [1.4–1.6]) | 1 (0.5% [0.1–2.8]) | -1.0 pp [-1.6–1.3] |
| Race/Ethnicity |  |  |  |
| Hispanic | 5622 (9.5% [9.3–9.8]) | 11 (5.5% [3.1–9.6]) | -4.0 pp [-6.7–0.3] |
| Non‑Hispanic White | 13702 (23.2% [22.9–23.6]) | 48 (24.1% [18.7–30.5]) | 0.9 pp [-4.9–7.6] |
| Non‑Hispanic Black | 36819 (62.4% [62.0–62.8]) | 131 (65.8% [59.0–72.1]) | 3.4 pp [-3.8–10.0] |
| Non‑Hispanic Asian | 284 (0.5% [0.4–0.5]) | 0 (0.0% [0.0–1.9]) | -0.5 pp [-0.5–1.5] |
| Other/Declined | 2545 (4.3% [4.2–4.5]) | 9 (4.5% [2.4–8.4]) | 0.2 pp [-2.1–4.2] |
| Employment |  |  |  |
| Full-time/Retired | 20940 (35.5% [35.1–35.9]) | 38 (19.1% [14.2–25.1]) | -16.4 pp [-21.7–-10.0] |
| Part-time | 4828 (8.2% [8.0–8.4]) | 11 (5.5% [3.1–9.6]) | -2.7 pp [-5.3–1.7] |
| Unemployed | 31887 (54.1% [53.7–54.5]) | 148 (74.4% [67.9–79.9]) | 20.3 pp [13.4–26.3] |
| Student | 701 (1.2% [1.1–1.3]) | 0 (0.0% [0.0–1.9]) | -1.2 pp [-1.3–0.8] |
| Unknown | 616 (1.0% [1.0–1.1]) | 2 (1.0% [0.3–3.6]) | -0.0 pp [-0.9–2.6] |
| Language |  |  |  |
| English | 57322 (97.2% [97.1–97.3]) | 197 (99.0% [96.4–99.7]) | 1.8 pp [-0.9–2.7] |
| Spanish | 1172 (2.0% [1.9–2.1]) | 2 (1.0% [0.3–3.6]) | -1.0 pp [-1.8–1.7] |
| Other | 478 (0.8% [0.7–0.9]) | 0 (0.0% [0.0–1.9]) | -0.8 pp [-0.9–1.2] |
| Marital Status |  |  |  |
| Single | 50847 (86.2% [85.9–86.5]) | 180 (90.5% [85.6–93.8]) | 4.2 pp [-0.9–7.9] |
| Married/Partner | 6126 (10.4% [10.1–10.6]) | 12 (6.0% [3.5–10.2]) | -4.4 pp [-7.2–0.1] |
| Divorced/Separated | 1570 (2.7% [2.5–2.8]) | 7 (3.5% [1.7–7.1]) | 0.9 pp [-1.1–4.5] |
| Other | 118 (0.2% [0.2–0.2]) | 0 (0.0% [0.0–1.9]) | -0.2 pp [-0.2–1.7] |
| Unknown | 311 (0.5% [0.5–0.6]) | 0 (0.0% [0.0–1.9]) | -0.5 pp [-0.6–1.4] |
| Primary Payer |  |  |  |
| Self‑Pay | 5777 (9.8% [9.6–10.0]) | 10 (5.0% [2.8–9.0]) | -4.8 pp [-7.3–-0.6] |
| Private | 11091 (18.8% [18.5–19.1]) | 16 (8.0% [5.0–12.7]) | -10.8 pp [-14.1–-5.8] |
| Medicare | 1154 (2.0% [1.8–2.1]) | 4 (2.0% [0.8–5.1]) | 0.1 pp [-1.3–3.2] |
| Medicaid | 40690 (69.0% [68.6–69.4]) | 169 (84.9% [79.3–89.2]) | 15.9 pp [9.9–20.6] |
| TRICARE/Military | 178 (0.3% [0.3–0.3]) | 0 (0.0% [0.0–1.9]) | -0.3 pp [-0.3–1.6] |
| International | 11 (0.0% [0.0–0.0]) | 0 (0.0% [0.0–1.9]) | -0.0 pp [-0.0–1.9] |
| Uninsured | 71 (0.1% [0.1–0.2]) | 0 (0.0% [0.0–1.9]) | -0.1 pp [-0.2–1.8] |

Note: Proportions are reported as n/N (percent [95% CI]). Absolute differences are reported in percentage points (pp) with 95% CIs.

In a retrospective, quasi-experimental EHR study across 20 EDs (2018–2023) of encounters among patients aged 15–44 years who received both pregnancy and GC/CT testing.

*ED*, emergency department; *pp*, percentage points; *CI*, confidence interval; *EHR*, electronic health record; *GC/CT,* gonorrhea and chlamydia

**Supplemental Table 4.** Demographic characteristics of pregnant ED patients with and without confirmed treponemal-positive syphilis serology

| Factor | Negative | Confirmed treponemal-positive |
| --- | --- | --- |
| Denominator (group totals) | N=5163 | N=15 |
| Age At Encounter | 24.0 [21.0, 29.0] | 30.0 [24.0, 34.0] |
| Homeless |  |  |
| Yes | 13 (0.3% [0.1–0.4]) | 0 (0.0% [0.0–20.4]) |
| Interpreter Needed |  |  |
| Yes | 119 (2.3% [1.9–2.8]) | 0 (0.0% [0.0–20.4]) |
| Race/Ethnicity |  |  |
| Hispanic | 574 (11.1% [10.3–12.0]) | 1 (6.7% [1.2–29.8]) |
| Non‑Hispanic White | 927 (18.0% [16.9–19.0]) | 5 (33.3% [15.2–58.3]) |
| Non‑Hispanic Black | 3397 (65.8% [64.5–67.1]) | 8 (53.3% [30.1–75.2]) |
| Non‑Hispanic Asian | 24 (0.5% [0.3–0.7]) | 0 (0.0% [0.0–20.4]) |
| Other/Declined | 241 (4.7% [4.1–5.3]) | 1 (6.7% [1.2–29.8]) |
| Employment |  |  |
| Full-time/Retired | 1694 (32.8% [31.5–34.1]) | 4 (26.7% [10.9–52.0]) |
| Part-time | 406 (7.9% [7.2–8.6]) | 0 (0.0% [0.0–20.4]) |
| Unemployed | 2963 (57.4% [56.0–58.7]) | 11 (73.3% [48.0–89.1]) |
| Student | 36 (0.7% [0.5–1.0]) | 0 (0.0% [0.0–20.4]) |
| Unknown | 64 (1.2% [1.0–1.6]) | 0 (0.0% [0.0–20.4]) |
| Language |  |  |
| English | 4958 (96.0% [95.5–96.5]) | 15 (100.0% [79.6–100.0]) |
| Spanish | 139 (2.7% [2.3–3.2]) | 0 (0.0% [0.0–20.4]) |
| Other | 66 (1.3% [1.0–1.6]) | 0 (0.0% [0.0–20.4]) |
| Marital Status |  |  |
| Single | 4454 (86.3% [85.3–87.2]) | 13 (86.7% [62.1–96.3]) |
| Married/Partner | 601 (11.6% [10.8–12.5]) | 0 (0.0% [0.0–20.4]) |
| Divorced/Separated | 88 (1.7% [1.4–2.1]) | 2 (13.3% [3.7–37.9]) |
| Other | 6 (0.1% [0.1–0.3]) | 0 (0.0% [0.0–20.4]) |
| Unknown | 14 (0.3% [0.2–0.5]) | 0 (0.0% [0.0–20.4]) |
| Primary Payer |  |  |
| *Self‑Pay* | 491 (9.5% [8.7–10.3]) | 1 (6.7% [1.2–29.8]) |
| *Private* | 803 (15.6% [14.6–16.6]) | 1 (6.7% [1.2–29.8]) |
| *Medicare* | 70 (1.4% [1.1–1.7]) | 0 (0.0% [0.0–20.4]) |
| Medicaid | 3784 (73.3% [72.1–74.5]) | 13 (86.7% [62.1–96.3]) |
| TRICARE/Military | 9 (0.2% [0.1–0.3]) | 0 (0.0% [0.0–20.4]) |
| Uninsured | 6 (0.1% [0.1–0.3]) | 0 (0.0% [0.0–20.4]) |

Note: Proportions are reported as n/N (percent [95% CI]).

In a retrospective, quasi-experimental EHR study across 20 EDs (2018–2023) of encounters among patients aged 15–44 years who received both pregnancy and GC/CT testing.

*ED*, emergency department; *CI*, confidence interval; *EHR*, electronic health record; *GC/CT*, gonorrhea and chlamydia

**Supplemental Figure 1.** Syphilis testing change among pregnant ED patients tested for both pregnancy and gonorrhea/chlamydia, 2018—2023

This quadrant analysis displays syphilis testing among pregnant ED patients who were tested for both pregnancy and gonorrhea/chlamydia. The plot compares each ED’s change in syphilis testing from 2018 to 2023 (y-axis) with its 2023 share of total testing volume (x-axis). Each point represents one ED; bubble size is proportional to the absolute increase in tests performed. Quadrants are defined by cohort-specific medians. Although overall volumes were smaller than among nonpregnant patients, several large EDs achieved substantial gains, accounting for most of the increase within this subgroup, in a retrospective, quasi-experimental EHR study across 20 EDs (2018–2023) of encounters among patients aged 15–44 years who received both pregnancy and GC/CT testing.

*ED*, emergency department; GC/CT, gonorrhea and chlamydia
